# Symmetric Biomechanically Guided Prone-to-Supine Breast Image Registration

**DOI:** 10.1007/s10439-015-1496-z

**Published:** 2015-11-17

**Authors:** Björn Eiben, Vasileios Vavourakis, John H. Hipwell, Sven Kabus, Thomas Buelow, Cristian Lorenz, Thomy Mertzanidou, Sara Reis, Norman R. Williams, Mohammed Keshtgar, David J. Hawkes

**Affiliations:** Department of Medical Physics & Biomedical Engineering, Centre for Medical Image Computing, University College London, Gower Street, London, WC1E 6BT UK; Philips GmbH Innovative Technologies, Research Laboratories Hamburg, Röntgenstrasse 24-26, 22335 Hamburg, Germany; Clinical Trials Group, Division of Surgery, University College London, Gower Street, London, WC1E 6BT UK; Department of Surgery, Royal Free Hospital, Pond Street, London, NW3 2QG UK; Division of Surgery, University College London, Gower Street, London, WC1E 6BT UK

**Keywords:** Image analysis, Image registration, Breast cancer, Biomechanics, Modelling, Finite difference method

## Abstract

Prone-to-supine breast image registration has potential application in the fields of surgical and radiotherapy planning, image guided interventions, and multi-modal cancer diagnosis, staging, and therapy response prediction. However, breast image registration of three dimensional images acquired in different patient positions is a challenging problem, due to large deformations induced to the soft breast tissue caused by the change in gravity loading. We present a symmetric, biomechanical simulation based registration framework which aligns the images in a central, virtually unloaded configuration. The breast tissue is modelled as a neo-Hookean material and gravity is considered as the main source of deformation in the original images. In addition to gravity, our framework successively applies image derived forces directly into the unloading simulation in place of a subsequent image registration step. This results in a biomechanically constrained deformation. Using a finite difference scheme avoids an explicit meshing step and enables simulations to be performed directly in the image space. The explicit time integration scheme allows the motion at the interface between chest and breast to be constrained along the chest wall. The feasibility and accuracy of the approach presented here was assessed by measuring the target registration error (TRE) using a numerical phantom with known ground truth deformations, nine clinical prone MRI and supine CT image pairs, one clinical prone-supine CT image pair and four prone-supine MRI image pairs. The registration reduced the mean TRE for the numerical phantom experiment from initially 19.3 to 0.9 mm and the combined mean TRE for all fourteen clinical data sets from 69.7 to 5.6 mm.

## Introduction

Breast cancer is the most common female cancer worldwide. The lifetime risk for a woman in Europe to develop breast cancer is estimated to be one in eight. Being diagnosed with breast cancer carries a high psychological burden for any patient and thus improved cancer management strategies are sought which streamline the clinical workflow and could potentially improve the clinical outcome whilst avoiding the risk of over-diagnosis.

When a woman is diagnosed with breast cancer, surgery is often part of her individual therapy plan which can also include additional forms of treatment such as chemotherapy and radiotherapy. Where possible *lumpectomy* combined with radiotherapy is the preferred treatment of choice. This involves removing only the cancerous tissue with a margin of healthy breast tissue, therefore conserving the unaffected parts of the breast. This has potential benefits over mastectomy, the complete removal of breast tissue, of being more acceptable to patients, offering good cosmetic results and comparably low risk of local recurrence.^26^

Early detection of breast cancer, for example through national screening programmes, identifies a significant proportion of lesions that might not be palpable. This is often the case if their size is below a diameter of 10 mm. For the surgical removal of such a lesion, strategies have to be in place to localise the cancerous tissue accurately without tactile guidance.^16^ Wire guided procedures are commonly used in these cases but radioactive seed localisation strategies have also been suggested.^22^

Once the cancerous tissue has been excised from the breast, the surgeon places marker clips into the cavity wall to provide information about the location of the tumour bed for subsequent radiotherapy. These clips are used to generate the radiation plan. The specimen is then sent for further histopathological examination to determine the presence, or otherwise, of cancerous tissue at the resection margin. Negative or positive margins are a strong predictor of local recurrence^2^ which may in turn influence the long-term survival.^17^

Routine, diagnostic, pre-surgical, dynamic contrast enhanced (DCE) MR images contain valuable information about the size and location of cancer in the pendulous, prone orientated breast. However surgery and radiotherapy are performed with the patient in the supine position. In this paper we describe a novel methodology for prone to supine, image-to-image registration. This has a number of potential applications:

- *surgical planning*, assuming an additional structural MR image has been acquired representing the approximate position of the patient in the operating room (OR),
- *initial pre-incision surgical guidance*, assuming the same supine MR image as above has been acquired and a methodology to transform the supine image into the physical coordinate system of the OR is also available (beyond the scope of this study), and
- *radiotherapy planning*, by relating the pre-operative MR image to a post-operative planning CT scan. The multi-modal aspect of this registration problem is covered in the current study, however, modelling of the tumour excision will also be required and is beyond the scope of this work.

Registration methods can broadly be classified into *image-to-image* and *image-to-physical-space* registration. The former class of algorithms—which is referred to as image registration for the remainder of this article—establishes a spatial correspondence between images. The latter registration type transforms image information to a real-world coordinate system, which makes it applicable for image guided procedures. Image guidance was first successfully established in the field of neurosurgery but was further expanded to orthopaedics, cardiac interventions, and thoraco-abdominal interventions, primarily using the assumption of rigid body alignment.^11^ In cases where the assumption of a rigid body motion does not apply, biomechanical models were employed to aid non-rigid alignment for instance due to brain shift^10^,^28^,^40^ or during liver surgery.^9^,^25^,^36^

Breast biomechanical modelling has been investigated by various research groups to aid different parts of the clinical workflow. In the early work of Azar *et al.*^3^ compression simulations with patient specific models using a piecewise linear approximation of non-linear tissue characteristics were carried out. These were later reported by Whiteley *et al.*^43^ as being inappropriate for simulating breast tissue deformations occurring in the prone and supine settings. Unloading the gravity loaded MR based geometry of the breast was addressed by Rajagopal *et al.*,^33^ as well as Pathmanathan.^32^ An overview of methods is presented in the review article by Babarenda Gamage.^5^ Surgical planning^15^ and guidance^7^ aims to provide pre-surgical image information to the surgeon. The major challenge to overcome in this task is that the position of the patient during pre-surgical imaging differs significantly from the surgical supine position. Alternative approaches regarding the patient positioning have been investigated but have not been adopted into clinical practice to date.^39^ Recently the potential role of supine MRI for image guided interventions was addressed by solving the image-to-physical-space registration task for which the comparably smaller deformation between the image acquisition and the surgical position was exploited.^1^,^12^ However, the authors concede that the lower image quality of the supine MR images may, in combination with the flattened breast geometry, make acceptance of this technology as a standard imaging procedure difficult.

Prone-to-supine image registration is to date an active topic of research.^4^,^8^,^20^,^23^,^29^,^30^,^35^ An overview is presented in Table 1. Rajagopal *et al.*^35^ and Babrenda Gamage *et al.*^5^ aim to solve this registration task with a pure biomechanical simulation approach. Their method uses a patient specific model, derived from prone MR images, to first remove the effects of gravity^33^ and subsequently reapply gravity loading into the supine direction. However, the assumption that only the direction of gravity changes from the prone imaging position to the supine surgical pose is an oversimplification. In addition, contact of the breast with the coil during the MR acquisition can introduce significant deformations which cannot be easily corrected using this method. In contrast Carter *et al.*,^8^ Lee *et al.*,^30^ Eiben et al.,^20^ and Han *et al.*^23^ also use a biomechanical finite element model to estimate the gravity induced deformation, but correct for the residual misalignment using a subsequent intensity based image registration step. However, intensity based registration methods that use generic transformation models might allow physically implausible deformations. Furthermore, some biomechanical simulations do not consider pre-stresses in the initial patient configuration.^23^,^29^ Motion of the breast tissue relative to the chest wall is also considered differently: While some models do not allow motion along the chest wall,^29^,^30^ others use prescribed displacements,^7^,^20^ frictionless sliding^23^, or an explicit model of the pectoralis muscle that also includes sliding.^5^ Defining appropriate prescribed displacements usually requires manual pre-processing which is undesirable in a clinical context. Lastly, a large variation in soft tissue elasticities might require an optimisation of the corresponding material parameters.^23^,^24^ In summary, a prone-supine registration approach that simultaneously: $\dagger 1$ considers pre-stresses in the initial patient position, $\dagger 2$ allows constrained motion of the breast tissue along the chest wall, $\dagger 3$ uses a biomechanically constrained deformation model $\dagger 4$ optimises the material parameters and $\dagger 5$ incorporates image information to correct residual misalignment, does not currently exist.

**Table 1:** Overview of published prone-to-supine registration methods.

Author	Simulation				Registration	
	Material type	Unloading	Element type	Chest motion	Deformation	Similarity
Rajagopal 2008^34^,^35^	Neo-Hookean	Inverse finite deform.^33^	Cubic Hermetian	Fixed	FEM	n.a.
Carter 2008^7^	Neo-Hookean	Iterative	Hexahedra	Prescribed	FEM+Fluid	NCC
Lee 2010^30^	Neo-Hookean	Not specified	Cubic Hermetian	Fixed	FEM+FFD	NMI
Babarenda Gamage 2012^4^	Neo-Hookean	Inverse finite deform.^33^	Cubic Hermetian	Fixed	FEM	n.a.
Lago 2012^29^	Mooney–Rivlin	Simple inversion	Not specified	Fixed	FEM	n.a. (surf. disp.)
Eiben 2013^20^	Neo-Hookean	Iterative	Tetrahedra	Prescribed	FEM+FFD	NMI
Han 2014^24^	Neo-Hookean	Simple inversion	Tetrahedra	Sliding	FEM+FFD	NMI

For the subsequent registrations, the transformation methods are either free form deformation (FFD) or finite element methods (FEM), where the similarity metrics are either normalised cross correlation (NCC) or normalised mutual information (NMI)

**Figure 1:**
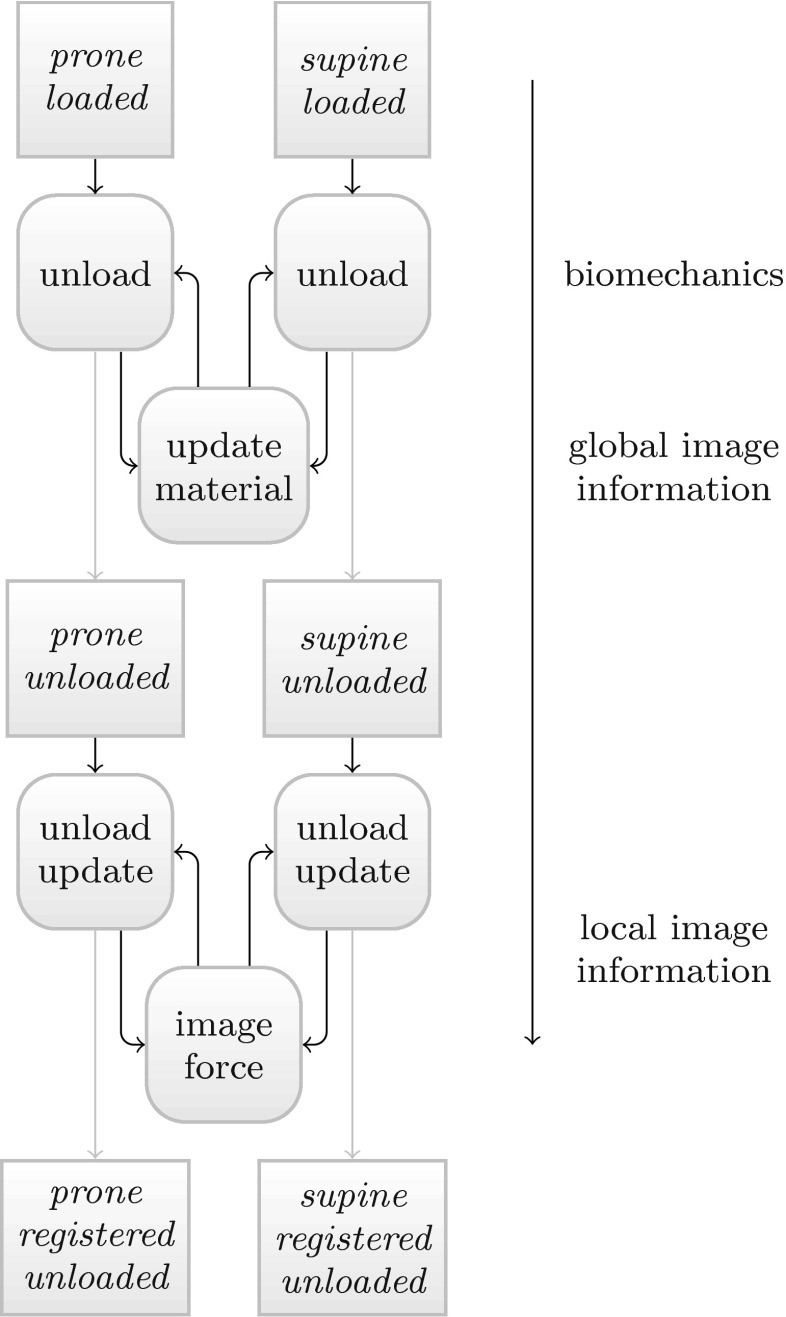
Overview of the biomechanics based registration procedure. In a first step the effect of gravity is removed from the prone and supine breast image assuming generic material parameters. In a second step the material parameters are repeatedly updated until the image similarity no longer improves. The final step involves activation of image derived forces which aim to correct modelling inaccuracies and generate the final aligned images in the unloaded configuration.

Our proposed method addresses these issues by integrating image registration components, i.e. image derived forces, directly into patient specific biomechanical simulations. Our symmetric, biomechanical image registration aligns the images in a central, virtually unloaded configuration and considers gravity as the main cause of pre-stresses in the breast as represented in the images. The algorithm is designed symmetrically, so that both the prone and the supine images are transformed simultaneously (c.f. Fig. 1). Hence the first step includes an unloading simulation, which only considers gravity as a body force $\dagger 1$,$\dagger 3$. Subsequently the alignment is improved by first updating the global material parameters $\dagger 3$,$\dagger 4$, and second by adding local image derived forces to the system $\dagger 3$,$\dagger 5$. These account for the residual misalignment and in turn update the unloaded configuration. This results in a biomechanically constrained deformation. We allow the breast tissue to move along the chest wall by implementing a tangential motion constraint in the retro-mammari area $\dagger 2$. By choosing a finite difference numerical solution scheme (FDM), we furthermore avoid the need for an explicit mesh generation step as required by finite element procedures. The implemented method is used to align prone-supine MR image pairs, and, to our knowledge, for the first time, prone MRI and supine CT breast images.

## Materials and Methods

### Overview of the Image Alignment Approach

The computational framework presented here is a symmetric, intensity based, biomechanically driven image registration method to align prone and supine breast images. It is a significant extension to our previous work^20^ and follows the idea that the main source of geometric deformation of the breast between the prone and the supine images arises from the relative difference in gravity loading. Thus when the effect of gravity is removed from the loaded breast configurations, and the images being transformed accordingly, the registration task becomes less challenging. The remaining dissimilarities arise primarily from modelling inaccuracies such as unknown material parameters, missing knowledge about the exact patient specific constitutive relation of* in-vivo* breast tissue and insufficient definition of boundary conditions due to contact with imaging equipment or undefined motion of the breast and muscle tissue on the chest wall.

Figure 1 shows an overview of the complete proposed algorithm. The main building blocks are:

- A biomechanical deformation model (“3D Finite Difference Simulation” section),
- the calculation of the patient-specific unloaded configuration (“Gravity Unloading” section),
- a tangential motion constraint to enforce the motion of the pectoral muscle along the chest surface (“Surface-Based Motion Constraint” section),
- a material update scheme (“Material Optimisation” section), and
- image derived forces (“Integration of Image Derived Forces” section).

The algorithm is designed to start with a pure biomechanical simulation but in the course of the execution image information is incorporated progressively directly into the unloading simulation: first on a global and then on a local scale.

### 3D Finite Difference Simulation

Constitutive relations and corresponding material parameters of breast tissues are open research topics. Samani* et al.*^37^,^38^ carried out mechanical tests on ex-vivo breast tissue samples. The mechanical properties of tissues however change significantly after removal from the* in-vivo* environment. In a recent study Eder *et al.*^19^ used a biomechanical finite element simulation based on prone MR images to simulate the breast shape in the upright standing position of a patient. The simulation was evaluated against surface scans of the same patients in the same position. They report that the material relations proposed by Tanner *et al.*^41^ and Rajagopal *et al.*^34^ produced the most accurate simulations. Interestingly both cited approaches use a simple neo-Hookean material constitutive relation. Hence the proposed image registration framework assumes breast tissues biomechanical description through this model, which requires only two material coefficients (Lamé parameters: $\lambda$ and $\mu$).^6^ This is an advantage if the knowledge about the exact material properties is limited either due to the lack of* in-vivo* measurements or incoherent literature values. Furthermore, in a clinical application scenario* in-vivo* measurements are usually not available.

The basis for the non-linear biomechanical deformation model is given by the principle of conservation of linear momentum:

1 $$\begin{aligned} \rho_0 \,\partial_{tt}{\mathbf{u}} = \nabla_0 \cdot {\mathbf{N}} + \rho_0 {\mathbf{f}} - r \partial_t {\mathbf{u}} \end{aligned}$$

Here $\mathbf{u}$ is the displacement vector, $\rho_0$ the mass density, *t* the time, $\mathbf{N}$ the nominal stress or the transposed non-symmetric first Piola–Kirchhoff Stress Tensor, $\mathbf{f}$ the body force and *r* the speed proportional damping coefficient. The first and second partial derivatives with respect to time are indicated by the operators $\partial_t$ and $\partial_{tt}$ respectively. The non-linear material response is defined by the nominal stress tensor $\mathbf{N}$ which, for the neo-Hookean model, is given by

2 $$\begin{aligned} {\mathbf{N}}_\text{NH} = \left( \mu \left( {\mathbf{I}} - {\mathbf{C}}^{-1}\right) + \lambda \ln (J) {\mathbf{C}}^{-1}\right) {\mathbf{F}}^\text{T}. \end{aligned}$$

The deformation gradient tensor $\mathbf{F}$ provides the measure of deformation between a point in the undeformed, $\mathbf{X}$, and a point in the deformed configuration, $\mathbf{x}$, and is defined by  while ${\mathbf{C}} = {\mathbf{F}}^{\text{T}}{\mathbf{F}}$ is the right Cauchy deformation tensor. The final quantity required to evaluate the material stress given in (2) is the volume change *J* which is the determinant of the deformation gradient: $J=\det (\mathbf{F})$.

Explicit time integration of Eq. (1) is obtained* via* a discrete central difference with respect to time which can be solved directly for the displacement at the next time step. The first and second time derivatives of the displacement vector field $\mathbf{u}$ can be approximated by the following forward and central differential operators

3 $$\begin{aligned} \partial_t {\mathbf{u}} \approx \partial_{t}^{+} U^{n}_{i, j, k}&= \frac{1}{h_t}\left( U^{n+1}_{i, j, k} - U^{n}_{i, j, k} \right) \end{aligned}$$

4 $$\begin{aligned} \partial_{tt} {\mathbf{u}} \approx \partial_{tt}^{\pm } U^{n}_{i, j, k}&= \frac{1}{h_t^2} \left( U^{n+1}_{i, j, k} - 2 U^{n}_{i, j, k} + U^{n-1}_{i, j, k} \right) \end{aligned}$$

where $$U^{n}_{i, j, k} $$ is the discrete version of the continuous and time dependent deformation vector field $${\mathbf{u}}({\mathbf{X}},t)$$ with spatial indices *i*, *j*, *k* corresponding to the position $$\mathbf{X}$$ and temporal index *n* corresponding to a point in time *t*.

Substituting the internal and external forces of (1) by $\mathbf{k}$ this equation can be rewritten:

5 $$\begin{aligned} \partial_{tt}^{} {\mathbf{u}} = {\mathbf{k}} -\frac{r}{\rho } \partial_{t}^{}{\mathbf{u}} \end{aligned}$$

Using the discrete time derivatives (3) and (4) and the appropriate discrete version of $$\mathbf{k}$$ denoted by $$K^{n}_{i, j, k} $$, an *explicit time integration scheme* is formulated by solving for $$U^{n+1}_{i, j, k} $$:

6 $$\begin{aligned} U^{n+1}_{i, j, k} = \frac{\left( 2\rho + h_t r \right) U^{n}_{i, j, k} - \rho U^{n-1}_{i, j, k} + h_t^2 \rho K^{n}_{i, j, k} }{\rho + h_t r} \end{aligned}$$

As this scheme is only conditionally stable, the critical step size has to be obeyed.

Similar to the discrete differential operators (3) and (4) which are defined with respect to time, discrete spatial derivatives can be formulated by substituting the time step $h_t$ with a spatial step $h_x, h_y, h_z$. The mixed spatial derivatives are required to solve (1) and can be approximated by the following differential operator:

7 $$ \partial_{xy}^{}{\mathbf{u}} \approx \partial_{xy}^{\pm }U^{n}_{i, j, k}= \frac{1}{4 h_x h_y} \left( U^{n}_{i+1, j+1, k} + U^{n}_{i-1, j-1, k} - U^{n}_{i-1, j+1, k} - U^{n}_{i+1, j-1, k} \right) $$

The spatial derivatives $\partial_{yy}^{}\mathbf{u}$, $\partial_{zz}^{}\mathbf{u}$, $\partial_{yz}^{}\mathbf{u}$, and $\partial_{xz}^{}\mathbf{u}$ follow by appropriate permutation of the discrete indices *i*, *j*, *k* in the equations above.

To complete the initial boundary value problem, we define an initial displacement of ${\mathbf{u}}_{t=0}={\mathbf{0}}$ and a homogeneous Dirichlet boundary condition on the boundary of the image domain.

### Gravity Unloading

One of the major assumptions made up to this point is that the geometry in the *unloaded* or *stress-free* state is known. Of course the equation of motion still holds true for pre-stressed objects as long as such stresses are added separately. However, measuring tissue pre-stressing in the context of* in-vivo* breast imaging is, to our knowledge, not feasible. Thus the stress-free breast geometry is unknown. However, the concept of the unloaded configuration permits reduction of the scale of the deformation problem at hand.

The method presented here applies the iterative prediction-correction scheme^8^,^20^,^21^ into the FDM framework. It uses the flexibility of the explicit time integration to recover the unloaded configuration in only one forward simulation by correcting the prediction during the course of the simulation. In the FDM framework the spatial material distribution of the involved compartments in the loaded configuration, namely chest, fat, gland, and background are directly related to the segmentation of the clinical MR or CT images. The loading aims to find the displacement vector field (DVF) which points from the unknown unloaded to the known loaded configuration as represented in the clinical images. Hence the DVF is determined by the biomechanical simulation and is unique for a given hyperelastic material configuration.

An overview of the developed unloading procedure using the example of the prone breast is given in the following: Starting with the geometry segmented from the prone loaded MR image, we build the biomechanical model and begin the forward loading simulation by applying gravity in the anterior direction. This means, that the material parameters $\mu (\mathbf{X})$ and $\lambda (\mathbf{X})$, the mass density $\rho_m(\mathbf{X})$ and body force ${\mathbf{f}}_m({\mathbf{X}})$ define the simulation at unloading step *m* and are initially for $m=0$ identical with the configuration shown in the clinical image

8 $$\begin{aligned} M_m({\mathbf{X}}):= \{\lambda_m({\mathbf{X}}), \mu_m({\mathbf{X}}), \rho_m({\mathbf{X}}), {\mathbf{f}}_m({\mathbf{X}})\}. \end{aligned}$$

The simulation itself describes the *forward mapping*$\varphi ({\mathbf{X}},n,M_m)={\mathbf{x}}$ of a material point $\mathbf{X}$. As a consequence of the application of gravity, the breast extends further anterior. Hence a correction of the basis of the biomechanical model—the interim unloaded configuration $M_m(\mathbf{X})$—is required: The loaded spatial material configuration as represented in the clinical images is pulled back—or warped—from the tip to the start of the vectors of the DVF, which means that the breast virtually contracts into the posterior direction, moving closer towards the estimated unloaded configuration.

9 $$\begin{aligned} M_{m+1}({\mathbf{X}}) = M_0(\varphi ({\mathbf{X}},n,M_m)) = M_0({\mathbf{x}}) \end{aligned}$$

In the field of image processing this step is known as resampling and is equivalent to the inverse mapping $\varphi ^{-1}:\mathbf{x}\mapsto \mathbf{X}$. With the updated unloaded configuration, the loading simulation is continued with repeated resampling steps at given time points. If the update of the material configuration becomes small such that $M_{m+1}\approx M_{m}$, the unloaded configuration has been recovered. This is the case, when the dynamic biomechanical simulation reaches a quasi-static state.

Inverting a deformation vector field to pull back $M_0$ into the current estimate of the unloaded configuration is usually an iterative and computational expensive procedure.^13^ However the backward Lagrangian perspective utilised in image transformation can be applied and inherently yields the inverse deformation. Thus the update procedure with the inverse displacement vector field simplifies to an image resampling or warping task known from image processing and efficient implementations can be used.

Updating the material configuration *M* is not required at each temporal simulation step *n*, since the incremental deformations are sufficiently small. Hence iteration steps *m* and *n* were kept separately. An evaluation of the unloading scheme can be found in the Appendix Unloading Evaluation.

### Surface-Based Motion Constraint

The flexibility of the explicit time integration scheme allows direct imposition of motion constraints or displacement updates on selected nodes. Such nodes are also sometimes known as *slave nodes*. This technique is used here to constrain nodes on the chest wall to only move tangentially along the chest surface.

One alternative to constrain the chest nodes to remain on the chest could be to use a prescribed displacement constraint on these nodes directly. This however is difficult and possibly error prone as a point-to-point correspondence on the chest between the prone or supine loaded configuration and the corresponding unloaded one is generally unknown. Furthermore due to a limited number of features on the retro-mammari region this patient specific correspondence cannot be established easily a-priori.

**Figure 2:**
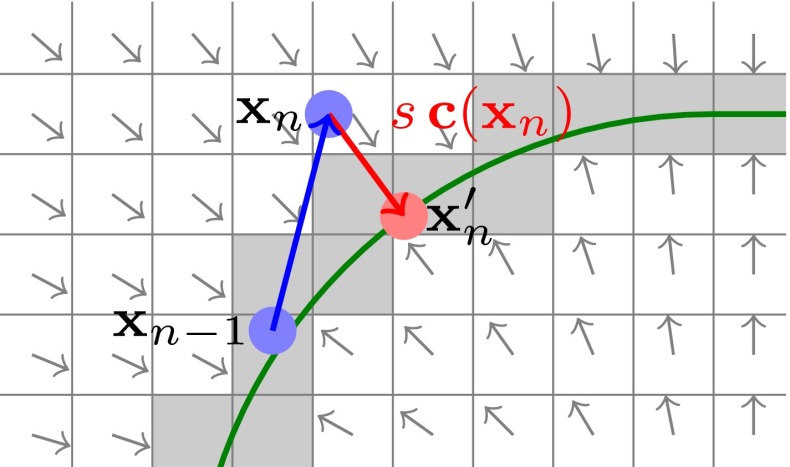
Surface based motion constraint. A discretised approximation of the target surface, depicted as grey shaded voxels, is generated from the chest-pectoral muscle interface based on the segmented image. From this a displacement vector field is pre-calculated which points to the target surface. This is used during the iterative solution process to displace nodes back to the target which due to the underlying material response might have moved out of this region.

In previous work, we assumed circumferential stretching when simulating the unloaded configuration from the prone and circumferential compression when simulating the unloaded configuration from the supine image.^20^ However, this assumption is an oversimplification of the underlying anatomy and the prescribed displacements compromise the alignment accuracy in this area directly. Thus a more flexible approach is followed here, where internal forces of the biomechanical simulation act as a regulariser for displacements parallel to the surface, whereas normal to the surface small correction displacements are applied. Figure 2 shows the general principle of our approach by depicting the course of a slave node during the simulation.

From the segmented prone MR and supine CT image, the target surface position of the chest is established and a corresponding Euclidean distance transformation is calculated. The gradient of the distance transform results in a correction vector field $\mathbf{c}(\mathbf{X})$. This then directs chest nodes, which during the course of the simulation move outside the target surface region, back to the surface. The chest-muscle boundary is extracted from the segmentation of the original prone and supine image and the corresponding nodes are labelled as chest or slave nodes. The deformed position of these nodes is then given by $\mathbf{x}_{\text{S}}$. During the course of the registration the position of these nodes is observed and corrected to

10 $$\begin{aligned} {\mathbf{x}}'_n = {\mathbf{x}}_n + s(v_{\text{s}})\,{\mathbf{c}}({\mathbf{x}}_n), \end{aligned}$$

if a node does not lie on the target surface. The scaling parameter $s(v_{\text{s}})$ controls the speed of the imposed surface alignment. This parameter has been chosen such that the explicit time integration converges. We here make the scaling dependent on the speed of a node towards the target surface $v_{\text{s}}$. When a slave node moves above a certain speed limit in the correction direction already, the amount of the correction will be decreased with a logistic function. We chose this function due to its smooth decrease above a specified value but expect that other functions with similar characteristics work equally well. The speed limit improves the stability of the dynamic system since repeated correction displacements accelerate the slave nodes which eventually might cause the system to diverge. The logistic function takes the form

11 $$\begin{aligned} s(v_{\text{s}}) =p \left( 1+e^{l(v_{\text{s}}-v_{\text{max}})} \right) ^{-1} \end{aligned}$$

with  and the constant correction parameter *p*. We observed that through the introduction of the speed dependent correction, the system became largely insensitive to the choice of *p*. We set $p=0.005$ and $v_{\text{max}} = 0.05\,{\text{m}}/{\text{s}}$ for all experiments.

Additional flexibility regarding the motion constraint can be achieved by varying the design of the correction vector field $\mathbf{c}(\mathbf{X})$. As specified in (10), either a tied surface boundary condition or a one-sided sliding condition can be imposed.

**Figure 3:**
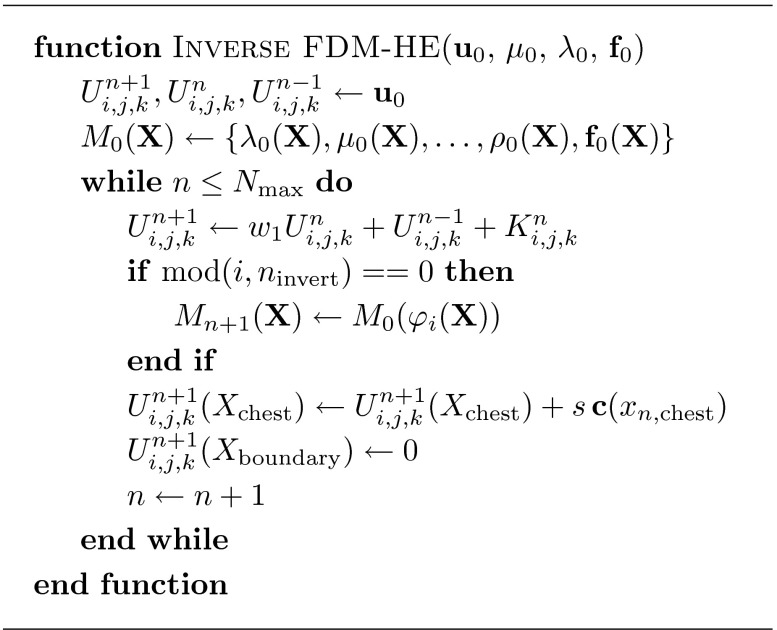
The base algorithm to calculate the unloaded configuration using a hyperelastic material, and the motion constraint for nodes on the chest wall.

Summarising the algorithm to this point, Fig. 3 shows an overview of our approach as described in detail in this and the previous section.

### Material Optimisation

Significantly different breast tissue stiffness has been reported in the literature. As a result, strategies to optimise the material parameters of a selected model have been investigated previously.^18^,^23^,^24^

We initialise our generic unloading simulation with an extremal material property—either very soft or stiff—and observe the influence of the changes to the material parameters on the image similarity measure (see “Integration of Image Derived Forces” section). If the similarity value improves, stiffening or softening steps are simulated until the image similarity does no longer improve. To cover a wider range of material parameters in less number of steps, a constant multiplicative factor is used to update the shear modulus repeatedly. This approach does not find the best possible parameters but a better starting position for the intensity based alignment.

### Integration of Image Derived Forces

In image registration an essential building block is a similarity or distance metric. But where in classical image registration only image forces act to align objects—usually counter balanced by a regularisation to obtain a smooth solution—here we consider the physical forces such as gravity as well as image forces simultaneously. The underlying hyperelastic material law acts as an implicit regulariser. Image forces lack physical meaning, but they are essential to drive the model in the direction required to align the images and thus help overcome modelling inaccuracies as described earlier.

For a mono-modal alignment task, the simplest and most widely used distance metric is the *sum-of-squared-differences* (SSD) which is defined as

12 $$\begin{aligned} \mathcal{S}_\text{SSD} := \frac{1}{2}\int_\Omega \left( P({\mathbf{X}})-S({\mathbf{X}})\right) ^2d{\mathbf{X}}. \end{aligned}$$

Here *P* and *S* denote the prone and supine image respectively that were warped into the current unloaded configuration $\mathbf{X}$. Since we are interested in aligning the prone to the supine unloaded image symmetrically, the forces need to be evaluated separately for prone and supine by formulating the Euler–Lagrange equation of (12), which gives^31^:

13 $$\begin{aligned} {\mathbf{f}}^{\text{SSD}}_{P}(X)&= -\left( P({\mathbf{X}})-S({\mathbf{X}})\right) \nabla P({\mathbf{X}}) \end{aligned}$$

14 $$\begin{aligned} {\mathbf{f}}^{\text{SSD}}_{S}(X)&= -\left( S({\mathbf{X}})-P({\mathbf{X}})\right) \nabla S({\mathbf{X}}) \end{aligned}$$

To incorporate the image forces into the simulation, the image forces are accumulated incrementally, i.e.,

15 $$\begin{aligned} {\mathbf{f}}_{\text{img}} = s(t) {\mathbf{F}}_N{\mathbf{f}}^{\text{SSD}}_{N} + \sum_{j=1}^{N-1} {\mathbf{F}}_j{\mathbf{f}}^{\text{SSD}}_{j}. \end{aligned}$$

Note, since the image is resampled from the loaded prone and supine configuration, the image forces are transformed by the deformation gradient $\mathbf{F}$ computed form the corresponding prone and supine unloading simulations. Furthermore the last evaluated image force is added using a polygonal loading function *s*(*t*) where $s(0)=0$ and $s(T)=1$. In order to keep a consistent record of the accumulated image forces, these are recorded in the loaded configuration, from which every quantity is subsequently resampled (see “Gravity Unloading” section).

**Figure 4:**
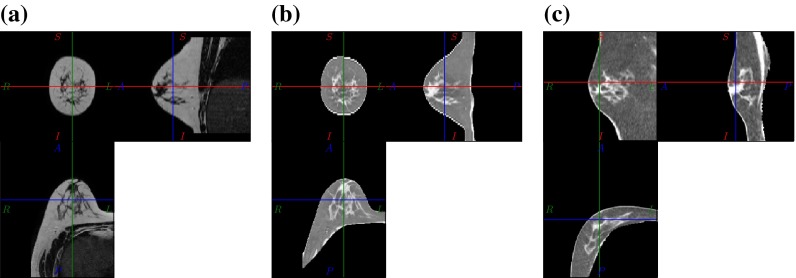
Intensity inversion of the prone T2 weighted MR image (a) is used to generate an image (b) which appears similar to a CT image (c) in terms of tissue contrast such that a mono-modal image similarity measure and corresponding image forces can be used.

The prone MRI to supine CT image registration task is obviously not of mono-modal nature. In this respect two different strategies could be followed. Either a multi-modal image similarity measure with corresponding image forces could be used, or one of the images is adapted so that the tissues appear with the same intensity as in the other modality. Here the latter approach was chosen since only two tissue classes are present and thus a simple intensity inversion with a linear scaling is sufficient. Namely the MR images are converted into pseudo CT intensities. An example for this intensity modification is shown in Fig. 4.

### Image Data

To evaluate the performance of the developed algorithm, different types of images were used.

Numerical phantom images were generated to allow evaluation of the registration quality with a known ground truth deformation over the entire virtual breast. The generation process of the numerical phantom dataset is described in detail in section “Numerical Phantom Datasets.”

The second set of image data consisted of nine prone MR and supine CT image pairs (P1–P9) which were acquired as part of the standard clinical procedure for breast cancer patients. The MR images were taken pre-operatively for diagnostic purposes and the CT images were post-operative planning CTs acquired just before radiotherapy. To avoid differences in the images caused by surgical tissue removal, we focus on the healthy contra-lateral breast.

We also added one prone-supine CT image data pair as a tenth patient (P10*) to the clinical data set. Since both images were acquired post-surgery, seven marker clips were present and well visible in both images. These clips are utilised to locate the tumour bed for radiotherapy in the clinical workflow. In the context of this study these clips could be used to generate a ground truth deformation between the two loading positions. To this end the seven markers were identified manually and warped according to the deformation vector field produced by the registration algorithm. The region of and around the markers was assigned with a registration mask so that no image forces were calculated here. Hence a bias in the registration result is avoided. Note however, that the clips are located only in the region of the original tumour location and thus cannot represent the registration accuracy for the entire breast. Furthermore four prone-supine MR image pairs were added to the clinical data set to allow a comparison of the registration performance between MRI–CT and MRI–MRI registration. A fifth dataset had to be excluded, since the field of view of the supine image was too narrow to use it as an input to our biomechanical registration.

To access and process the data, approval of the local ethics committee was obtained and the study was approved by the research and development unit of the clinical site. The T2 weighted MR images of the MRI–CT datasets have a native resolution of $0.625\times 0.625 \times 3\,{\text{mm}^3}$ and the CT images of $1.07\times 1.07 \times 3\,{\text{mm}^3}$. The prone MR images of the MRI image pairs have a native resolution of $0.7\times 2.2 \times 0.7\,{\text{mm}^3}$ and the corresponding supine images one of $0.7\times 0.7 \times 2.5\,{\text{mm}^3}$.

Processing of the images involved resampling to an isotropic resolution of $1\times 1 \times 1\,{\text{mm}^3}$, a bias-field correction of the T2-weighted MR images and the segmentation of both modalities into background, chest, fibro-glandular and adipose tissue with our in-house algorithm, which first determines the patient outline and then the pectoralis-breast boundary. This area is further segmented with an expectation maximisation algorithm into fat and fibro-glandular tissue. As a last step the chest wall of the supine image was manually rigidly aligned to the chest of the prone image.

### Numerical Phantom Datasets

In order to assess the feasibility and accuracy of the developed algorithm in a controlled environment, a numerical phantom dataset was generated. Ground truth deformations were generated using a finite element simulation, against which the registration could be compared. Note, that the simulated deformations are an idealisation of the deformations expected for the clinical cases.

**Figure 5:**
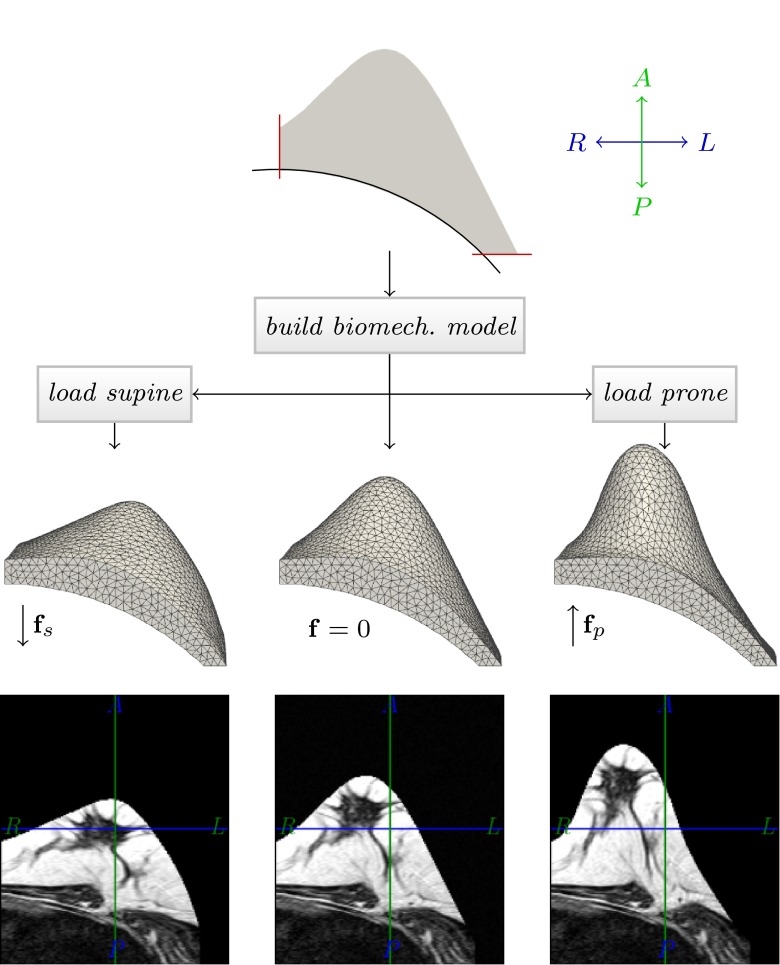
A simplified geometric numerical phantom was used to evaluate the performance of the presented algorithm. A cylinder represents the chest wall (black line in first row) and the skin is given by the function value of a two-dimensional Gaussian function (grey area over the cylinder section). A mesh of this geometry is generated and the biomechanical finite element model is built to simulate the effect of prone and supine gravity loading. The simulated prone and supine displacements are then used to transform the glandular structure of an MR image into the prone and supine position.

To approximate the geometry of a breast in the unloaded configuration, the surfaces of the chest wall and the skin were approximated by simple geometric forms. The chest wall was defined by a cylinder with its axis resembling the cranio-caudal patient axis. To define the skin surface, the height of a two-dimensional Gaussian function was added to the anterior elevation of the cylinder as shown in the first row of Fig. 5. The boundaries were defined as axial coronal and sagittal planes and set to fixed boundary condition for the FE simulations. This geometry was meshed and a biomechanical model with homogeneous neo-Hookean material properties was generated. Gravity was added as a body force acting on the unloaded configuration in the anterior and posterior directions to simulate the prone and supine gravity loaded configurations using *NiftySim*.^27^ The parameters of the geometry were chosen such that the numerical phantom geometry was comparable to a medium sized breast in terms of volume, extent and chest diameter. The left-right, anterior-posterior and superior-inferior extent of the numerical phantom were 160.4, 137.8, and 159.5 mm respectively and the enclosed volume was 1.14 L.

From the unloaded and simulated geometries corresponding images were generated by assigning the image texture of an MRI breast dataset to the unloaded geometry and warping it according to the simulated displacements.

## Results

### Numerical Phantom Registration

In order to quantify the performance of the registration algorithm in a controlled setting with known ground truth, the simulated prone and supine phantom images were registered using the proposed algorithm. The chest wall was assigned with a prescribed zero-displacement condition as the motion constraint used in the registration was not available in the finite element simulations. The registration was performed with an isotropic simulation grid spacing of $\Delta x_{\text{sim}} = \Delta y_{\text{sim}} = \Delta z_{\text{sim}} = 9.07\,{\text{mm}}$ and an image similarity or force resolution of $\Delta x_{\text{img}} = \Delta y_{\text{img}} = \Delta z_{\text{img}} = 2.27\,{\text{mm}}$. The critical time step of the explicit time integration scheme depends on the mesh density and thus a relatively coarse grid was chosen for the purpose of acceptable computational times. The image forces were calculated two levels finer than the simulation itself and transferred to the coarser resolution level to update the simulated unloaded configuration. The material update performed three stiffening steps with a factor of 1.2 for the parameters $\mu$ and $\lambda$, and is terminated when a decrease in the similarity was detected, while the final unsuccessful update is discarded.

**Figure 6:**
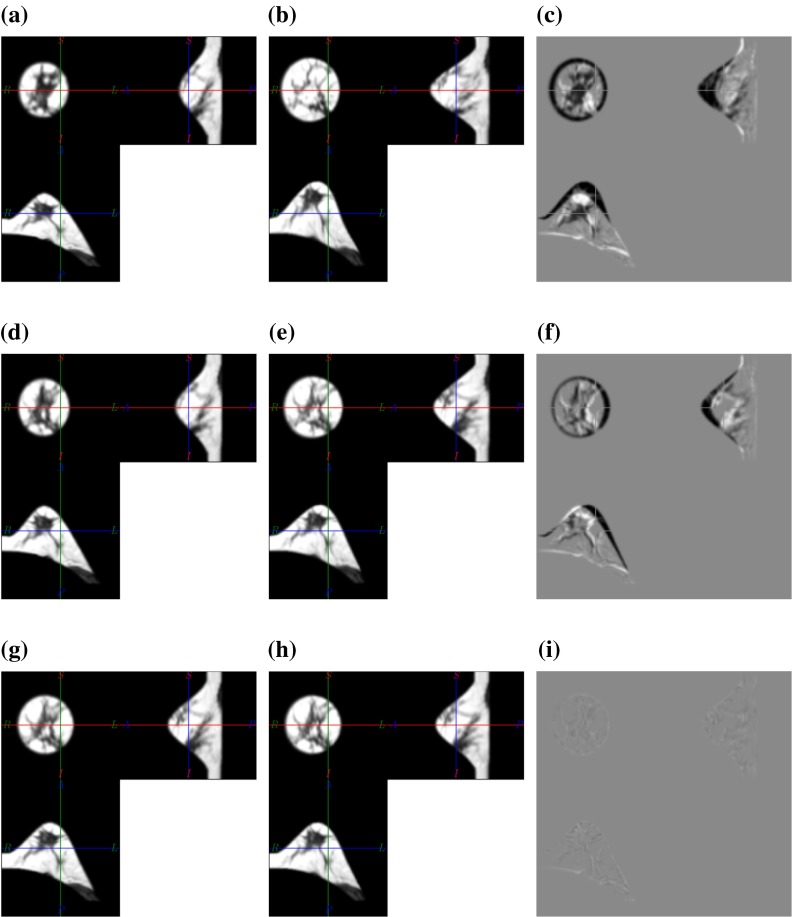
Registration results for the simulated prone and supine images. The first column (a, d, g) shows the state of the prone image during the course of the registration procedure, the second column (b, e, h) the corresponding states of the supine image and the third column (c, f, i) the difference images. The first row represents the warped images after the unloading procedure with generic material parameters (figures (a) and (b) show the initial unloaded prone and supine images respectively). Obviously the material parameters were chosen to be too soft and thus were iteratively stiffened to obtain a better match in the unloaded configuration (figures (d) and (e) show the unloaded prone and supine images after material parameter optimisation respectively). The alignment was then improved by accumulating image forces leading to the results shown in the third row. The difference images (c, f, i) are scaled so that the intensity range is equal for all difference images.

Figure 6 shows the intermediate and final results of the numerical phantom registration experiment. One can observe, that the initial material parameter estimates were indeed incorrect as the prone and supine images deform beyond the unloaded state (compare Figs. 6(a), 6(b), and 6(c) with Fig. 5). After the material update step the alignment was significantly improved, but not ideal. This can be attributed to the coarse material optimisation steps as well as to the coarse simulation resolution. This step provides a better starting point to achieve the final alignment. To this end image forces were accumulated to update the unloaded configurations accordingly. The final alignment is visually excellent as can be seen in the difference image (Fig. 6i). Furthermore the recovered unloaded configuration coincides with the initial one.

The target registration error (TRE) was evaluated for 500 landmarks randomly distributed across the initial numerical phantom domain. The quantitative results are given in Table 2. The mean TRE was reduced from $19.3\pm 16.2$ to $0.9 \pm 0.8\,{\text{mm}}$. Note that the relatively large maximum error of 6.1 mm after registration occurred at the border of the model, where the boundary conditions of the finite element simulation and the finite difference framework were not equivalent. In order to allow more deformation at the image borders, in the registration framework we apply padding around the image before registration. This differs from the ground truth deformation and the absence of image information in this region explains this behaviour. However, the initial maximum TRE was reduced by an order of magnitude.

**Table 2:** Target registration error of the registration of the numerical phantom dataset.

Registration step	TRE [mm]		
	Mean	Std.	Max.
No registration	19.3	16.2	58.7
Unloading, generic material	11.6	5.5	26.6
Material updated	5.4	2.9	20.1
Image forces	0.9	0.8	6.1

### Prone to Supine Registration of Clinical Data

In order to align prone MR images to the corresponding supine CT images (P1–P9), the intensities of the MR images were modified such that the grey values of fat and glandular tissue appeared similar in both images. To achieve this the intensity inversion within the breast segmentation mask was applied as described in section “Integration of Image Derived Forces.” Furthermore we selected a region of interest which contained the breast that was not operated to avoid effects of tissue removal between the images. The supine CT image was then manually rigidly aligned on the chest wall and sternum using the costal cartilage and adjacent rigid structures visible in both modalities. The prone-supine CT image pair (P10*) and the four MR image pairs (M1–M4) were processed in the same way except for the modification of the image intensities.

The image registration was performed with three progressively finer image resolution levels with $\Delta x_\text{img}= \Delta y_\text{img}=\Delta z_\text{img}=\{4,2,1\}\text{mm}$ following the well established methodology of multi-scale registration. The simulation level was kept at a constant isotropic resolution of $\Delta x_\text{sim} = \Delta y_\text{sim}= \Delta z_\text{sim} = 8\,{\text{mm}}$. Initial sensitivity experiments with twice the resolution of the simulation grid resulted in near identical registration results with differences at the scale of the voxel resolution. The difference in displacement measured $2.2\,{\text{mm}}\,(\pm 0.8\,{\text{mm}})$. For computational efficiency therefore we decided not to choose a finer grid for the registrations. Furthermore, no correlation between registration error and breast size could be identified.

Manually picked landmarks were used for the evaluation of the alignment quality in the central configuration. For each case eight to fourteen landmarks were selected first by one observer, where local adipose-to-fibroglandular tissue contrast allowed corresponding features in the prone and supine images to be identified. The left part of Table 3 (“Single observer”) shows the corresponding TREs for all evaluated cases. Since the images were aligned rigidly, the landmark distance before the registration allows measurement of the scale of the tissue motion between prone and supine positions. The mean landmark distance between the unregistered prone and supine positions, for all cases, ranges between 38.8 and 133.1 mm and the maximum landmark distance between 56.4 and 154.2 mm.

**Table 3:** Target registration error of the registration of the clinical MRI–CT datasets (P1–P9), the CT-CT dataset (P10*) and the MRI–MRI datasets (M1–M4) using manually selected landmarks.

	Single observer									Two observers, combined								
	Rigid			Unloading			Image forces			Rigid			Unloading			Image forces		
	Mean	Max.	Std.	Mean	Max.	Std.	Mean	Max.	Std.	Mean	Max.	Std.	Mean	Max.	Std.	Mean	Max.	Std.
P1	63.7	83.3	20.9	15.4	30.7	5.8	6.2	10.8	2.8	62.4	80.6	20.5	15.0	30.7	5.6	6.5	12.8	3.1
P2	58.7	88.5	17.1	16.1	24.4	7.1	8.0	12.8	3.4	61.5	88.5	17.9	14.3	24.1	6.6	7.0	12.8	3.3
P3	90.2	109.3	12.6	13.4	18.3	3.2	4.5	11.5	3.4	95.8	110.7	8.6	14.7	22.9	3.1	5.0	16.6	4.2
P4	93.9	131.3	20.2	19.9	33.9	7.1	9.6	28.6	7.2	98.4	134.9	22.7	22.5	35.3	7.1	9.1	20.2	5.3
P5	38.8	56.4	11.9	12.2	20.5	4.5	7.2	11.5	3.5	35.7	51.2	11.6	11.1	20.5	4.6	6.1	13.2	3.4
P6	50.6	58.4	7.7	6.5	11.9	3.3	4.8	10.5	2.6	49.1	58.6	9.9	7.5	12.4	3.0	4.2	9.1	2.0
P7	54.3	76.8	16.2	12.3	31.2	8.6	8.7	18.2	5.5	55.0	76.9	14.7	12.7	31.2	9.9	8.5	18.6	6.6
P8	91.8	125.7	25.8	24.3	34.4	5.8	5.6	13.9	3.7	101.2	125.7	18.2	25.4	35.0	5.9	5.4	12.3	2.9
P9	62.1	87.6	22.2	16.5	29.4	7.4	7.7	15.0	3.9	54.7	87.6	25.0	18.9	30.3	7.4	8.3	15.6	4.3
P10*	133.1	154.2	18.9	14.6	27.8	8.5	5.4	22.1	5.4	134.0	151.3	17.5	13.3	27.1	7.9	4.0	8.2	2.3
M1	54.7	67.7	7.2	10.3	22.0	5.2	3.1	6.9	1.9	59.4	67.7	4.5	9.2	16.7	4.2	3.0	4.3	1.1
M2	48.2	70.4	11.7	9.5	24.0	5.1	3.8	13.1	2.9	47.7	70.4	12.4	9.6	24.0	5.2	4.1	13.1	3.0
M3	51.8	61.1	6.9	11.9	19.7	4.4	4.5	6.7	1.2	49.9	64.4	7.4	11.3	16.6	4.1	4.6	8.5	1.8
M4	70.6	80.3	7.0	12.6	17.1	3.2	2.9	5.3	1.6	70.6	80.3	6.8	12.5	17.3	3.3	2.9	6.0	1.6
P1–P10*	73.7			15.1			6.8			74.8			15.5			6.4		
M1–M4	56.3			11.1			3.6			56.8			10.6			3.7		
P1–M4	68.7			14.0			5.9			69.7			14.1			5.6		

All values are given in mm

A significant reduction in the TRE can be observed by performing the unloading simulation and material optimisation. This results in an overall mean TRE of 14.0 mm varying between 6.5 and 24.3 mm and a maximum TRE between 11.9 and 34.4 mm. The number of successful material update steps that improved the image similarity as well as final material parameters for all cases are given in Table 4. Subsequently the final alignment was calculated by refining the unloaded configuration by adding image forces to the system. This resulted in a final overall mean TRE of 5.9 mm varying between 2.9 and 9.6 mm and maximum TREs between 5.3 and 28.6 mm. The final mean TRE for the MRI cases (M1–M4) is with 3.6 mm smaller than the 6.8 mm achieved for the cases P1–P10*.

**Table 4:** Number of accepted material optimisation steps, $N_{\text{opt}}$, each of which reduced the initial shear modulus by 10%.

Case	P1–P4	P5	P6	P7-P8	P9	P10*	M1–M3	M4
$N_{\text{opt}}$	0	5	2	0	8	4	0	2
$\mu\,[\text{Pa}]$	357.1	221.8	295.2	357.1	166.6	243.9	357.1	295.2

Only steps that improved the image similarity were accepted. The final shear modulus $\mu$ for each case is given in the bottom row

**Figure 7:**
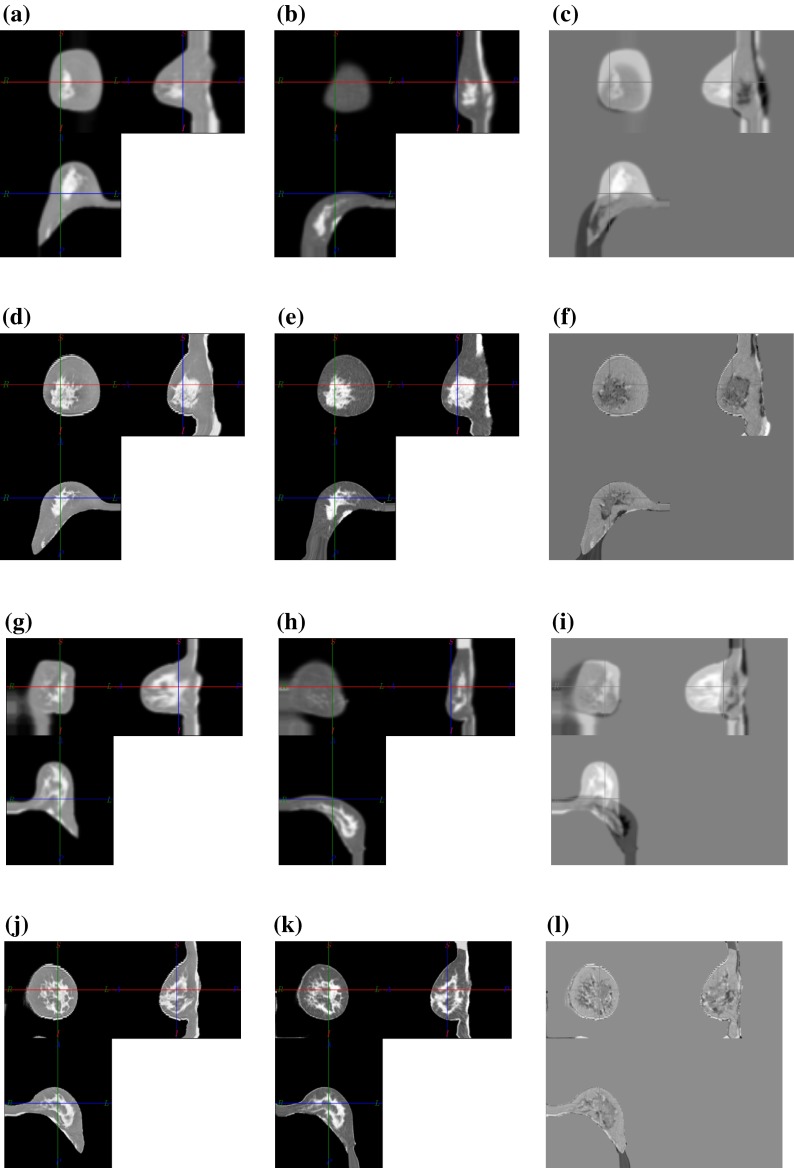
Orthogonal slices through the initial images and registration result for the lowest and highest target registration error reported in Table 3. The first column shows the prone, the second the supine and the third the corresponding difference images. The first two rows show P3 for which a landmark distance of 4.4 mm was measured, whereas the lower two rows show P4 where a mean landmark distance of 9.6 mm was measured after registration.

The results for the lowest and highest final landmark difference achieved for the MRI–CT cases, namely P3 and P4, are shown in Fig. 7. A good alignment of internal structures can be observed in both cases, however the residual alignment error actually appears to be higher for P4 in the difference image Fig. 7(i).

**Figure 8:**
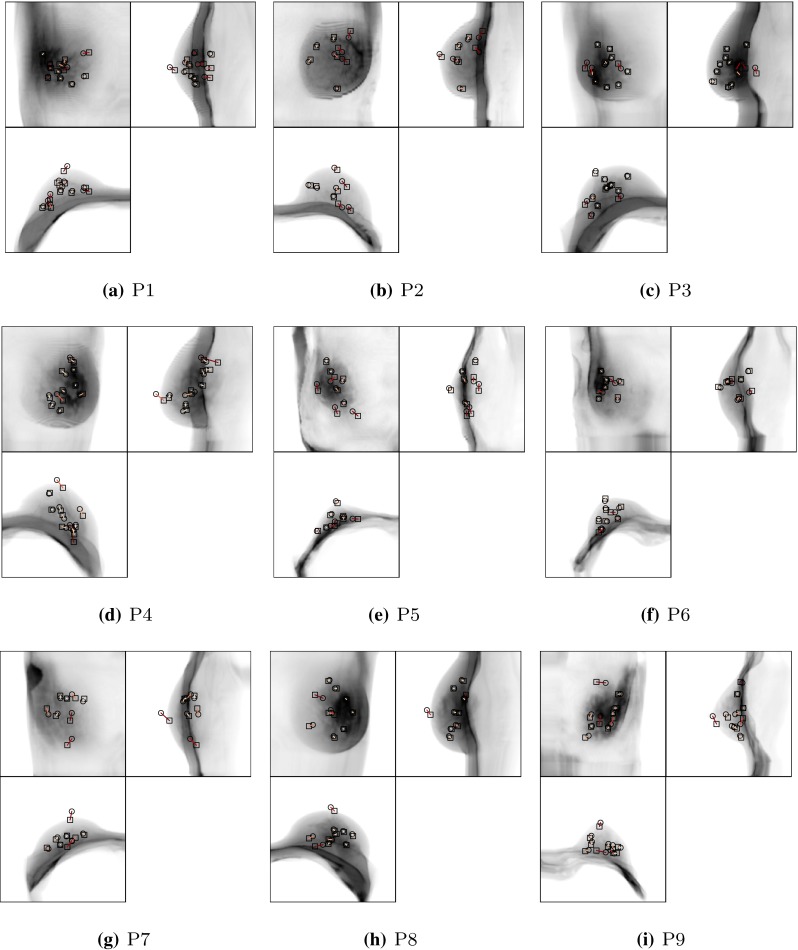
Visualisation of the target registration error in terms of landmark positions in the central virtually unloaded configuration. Landmarks were manually picked and transformed from the prone (circles) and supine (squares) position according to the registration result. To provide an aid of localisation the coronal, sagittal and axial mean intensity projection of the unloaded prone image are shown as grey-scale images.

Figure 8 shows the landmark distance in the central configuration as projections into the coronal, sagittal and axial planes for all evaluated MRI–CT cases. The landmarks transformed from the prone position are depicted as circles whereas those transformed from the supine position are shown as small squares. The correspondence is visualised as connecting lines, and the colour indicates the total Euclidean landmark distance. This allows a visual assessment of the distribution of the selected landmarks and of the registration accuracy throughout the breast.

**Figure 9:**
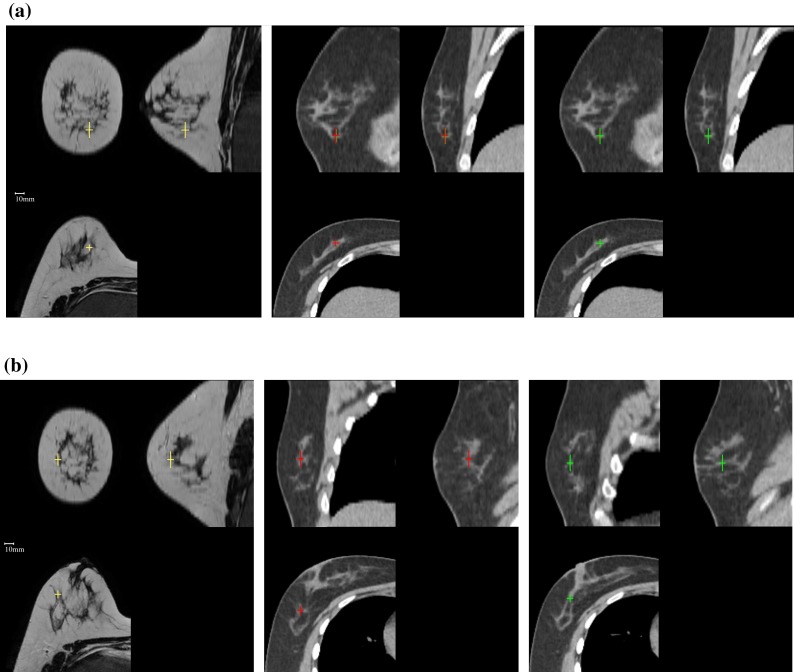
Orthogonal sections through the prone MRI and supine CT image of case P1 with corresponding landmarks selected by two observers. The first observer selected corresponding points in the prone and supine images (left and centre), whereas the control observer was asked to find the supine landmark when presented with the prone one (right). (a) shows an excellent agreement between the two observers (red and green crosses in the supine CT) resulting in a landmark distance of 1.1 mm. (b) is an example where both observers do not agree, identifying different structures with similar appearance resulting in a landmark distance of 15.2 mm.

For a clinically applicable registration quality, a registration error below 10.0 mm is desirable. This figure was obtained following discussions with clinicians. The mean TRE fulfils this criterion, but the maximum TRE of 28.6 mm does not. However, the landmark selection process is inherently observer dependent and potentially error prone, hence in a second step the quality of the landmarks was assessed by a second *control observer* with the aim to eliminate unreliable landmarks. This control observer was presented with the prone landmarks only and then given the task of selecting the corresponding landmarks in the supine image. The results of this inter-observer variability experiment are given in Table 5. Two examples of the 149 landmarks are shown in Fig. 9. The first example (Fig. 9a) shows a very good agreement between the observers, which is reflected in a landmark distance of 1.1 mm. Figure 9b on the other hand shows poor agreement between the observers, apparently due to visually similar structures. The landmark distance for this case is 15.2 mm. Eliminating such landmarks increases the confidence in the remaining landmarks to better reflect the actually achieved TRE.

**Table 5:** Inter observer variability in the supine configuration before and after exclusion of unreliable landmarks.

	Inter observer distance			Distance after exclusion			NL	NO
	Mean	Max.	Std.	Mean	Max.	Std.		
P1	5.1	15.2	3.9	4.2	10.0	2.5	12	1
P2	5.7	15.1	4.8	3.1	7.3	1.9	8	2
P3	7.3	24.6	8.2	3.0	9.2	2.3	13	3
P4	9.6	29.9	9.2	4.7	7.2	1.6	11	3
P5	6.7	21.6	6.2	3.9	8.5	2.4	9	2
P6	8.3	18.3	4.8	5.4	8.9	2.3	9	3
P7	7.6	15.2	4.7	5.5	9.6	3.5	8	2
P8	7.0	21.7	5.9	4.5	9.7	2.7	10	2
P9	7.6	16.5	6.0	2.8	4.8	1.3	10	4
P10*	8.5	29.8	9.5	3.6	9.2	2.4	13	3
M1	10.9	22.1	7.1	4.0	6.0	1.4	11	6
M2	5.7	28.6	7.0	3.3	8.9	2.1	14	2
M3	7.2	16.4	5.1	3.6	4.7	0.8	11	4
M4	1.8	2.9	0.8	1.8	2.9	0.8	10	0
P1–P10*	7.3			4.1				24.3%
M1–M4	6.4			3.2				26.3%
P1–M4	7.1			3.8				24.8%

*N*_L_ is the total number of landmarks and *N*_O_ the number of outliers. In the last three rows the relative number of landmarks which were above a threshold of 10 mm is given. Such landmarks were regarded as unreliable and excluded from the evaluation.

**Figure 10:**
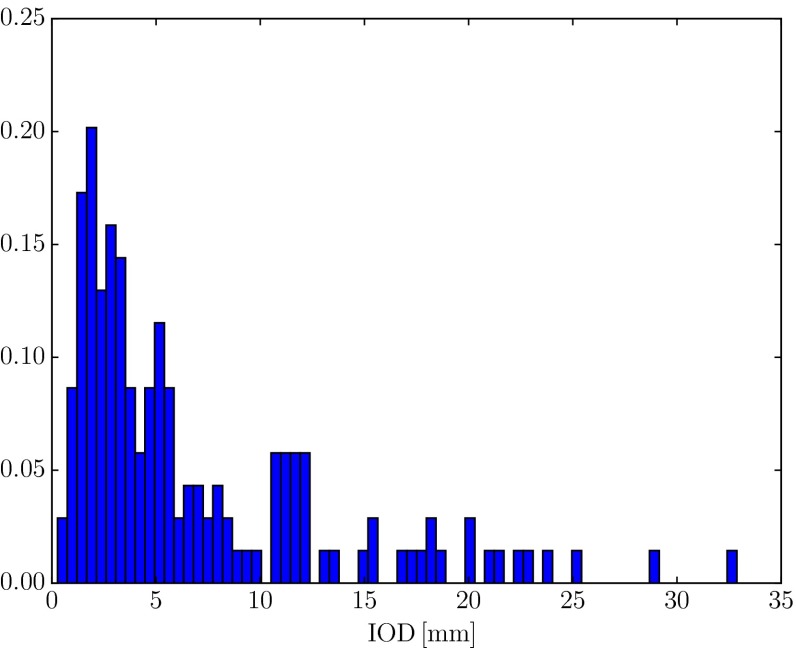
Histogram of all inter-observer distances.

Landmarks for which the distance between the first and the control observer were larger than 10 mm were eliminated from the evaluation. Visual inspection of the statistical distribution of all inter-observer distances as shown in Fig. 10 suggests a mixed distribution, with a cluster of values centred around 3.5mm and a distinct drop at 10 mm. Furthermore with increasing inter-observer distance, the chance that different structures within the breast were identified increases. For this reason 10 mm was taken to be a plausible distance, above which two landmarks can be considered placed on different features (see also 10 mm mark in Fig. 9). The landmarks of the second observer that were within 10 mm of the first observer were then added to the evaluation.

The overall mean inter-observer distance was 7.1 mm before and 3.8 mm after the exclusion of the outliers. The inter observer distance for the cases P1–P10* is 7.3 mm and only slightly higher than the distance measured for the cases M1–M4, which is 6.4 mm. For both groups, P1–P10* and M1–M4, about a quarter of the landmarks were excluded. This suggests, that there is a negligible difference in the accuracy of the landmark selection between MRI–MRI and MRI–CT cases. Accordingly, the right-hand side of Table 3 (“Two observers, combined”) shows the registration evaluation for the trusted landmarks only. The maximum TRE alignment error of 28.6 mm observed for P4 was reduced to 20.2 mm, whereas the overall mean registration error slightly reduced to 5.6 mm.

**Figure 11:**
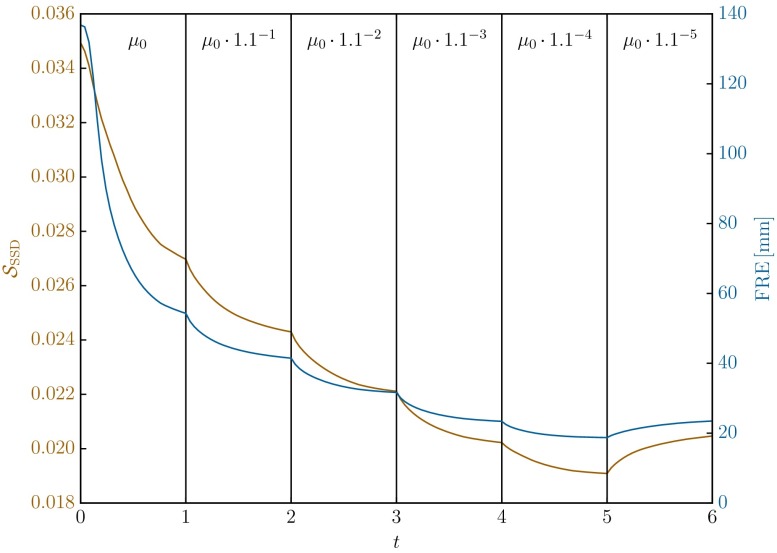
Image similarity measure, $\mathcal{S}_\text{SSD}$, and the corresponding registration error, $\text{FRE}$ (of the implanted fiducial markers), for case P10* over the course of the material optimisation iterations. After the unloading simulation with the generic material parameters $(0\rightarrow 1)$, five softening steps were performed $(1\rightarrow 6)$, each of which decreasing the material parameter value by about 10%. The fifth step $(5\rightarrow 6)$ resulted in an increased $\mathcal{S}_\text{SSD}$ and was rejected. Note that the FRE is not used to control the optimisation and only plotted to emphasise the validity of the similarity measure as a surrogate for the actually achieved alignment quality.

West *et al.*’s highly cited seminal paper from 1997^42^ showed that sub-voxel (and sub-millimetre for the data sets used) target registration accuracies could be achieved using intensity based CT to MRI registration methods. This ideal is dependent upon a number of factors which considerably reduce the likelihood of achieving such an accuracy for the prone-to-supine breast image application considered here. In particular the TREs reported in Ref. 42 were measured against an accurate gold standard reference transformation obtained using skull implanted fiducial markers; our gold standard is provided by the considerably less accurate manual identification of corresponding landmarks in the two modalities. Further, prone and supine breast images differ by a large non-rigid transformation, whereas the neurosurgical data sets in Ref. 42 could be accurately aligned using a rigid-body transformation. However, in addition to the manually selected landmarks as presented in Table 3, implanted fiducial markers could be used to evaluate the registration accuracy for the CT-CT case P10*, without inter-observer variability but only for a small region of the breast. In the prone configuration the axis aligned bounding box enclosing the seven landmarks measured $11.0 \times 16.6\times 10.1\,{\text{mm}^{3}}$. The rigid alignment of the images on the chest resulted in a mean (maximum) fiducial registration error (FRE) of 136.9 mm (139.6 mm). After the unloading and material optimisation the mean (maximum) FRE was reduced to 18.7 mm (22.4 mm). The final mean (maximum) FRE with accumulated image forces measured 3.61 mm (5.13 mm). The fiducial markers present in this case were also used to validate the material optimisation with repeated update steps. To achive this, the image similarity measure as well as the FRE were measured during the course of the optimisation. The result is shown in Fig. 11. It demonstrates that the image similarity used to control the optimisation procedure, is a valid surrogate for the alignment accuracy.

Last but not least the sensitivity of the final registration accuracy with respect to the initial rigid alignment is investigated. For this experiment the dataset P10* was selected since this case represents a clinical case with the additional benefit of implanted fiducial markers as detailed above. For the sensitivity analysis, we repeated the registration for this case with artificial translational offsets of 5 and 10 mm that were added to the translational component of the initial rigid alignment, namely $t_x$, $t_y$, and $t_z$. This results in a misalignment prior to registration in the left-right (LR), anterior-posterior (AP), and superior-inferior (SI) direction respectively. Table 6 summarises the registration accuracies using the implanted fiducials, as well as the combined landmarks. It can be observed that a misalignment of 5 mm has a negligible influence on the registration accuracy, as has a misalignment of 10 mm in the LR and AP direction. Only a translation of 10 mm in the SI direction decreases the registration accuracy slightly more, however the increase of about 3 mm is still well below the original displacement. Hence it can be concluded, that the registration is within limits insensitive to the initial alignment.

**Table 6:** Sensitivity of the fiducial and target registration error with respect to artificial left-right (LR), anterior-posterior (AP) and superior-inferior (SI) displacements which were added to the initial rigid transformation for case P10*.

	Fiducials			Two observers		
	Mean	Max.	Std.	Mean	Max.	Std.
Baseline	3.6	5.1	1.2	4.0	8.2	2.3
LR: *t*_*x*_ + 5 mm	3.5	4.3	0.8	4.0	8.7	2.2
LR: *t*_*x*_ + 10 mm	3.2	4.3	0.7	3.8	8.3	2.1
AP: *t*_*y*_ + 5 mm	3.7	4.9	1.0	3.7	7.4	2.0
AP: *t*_*y*_ + 10 mm	3.9	5.8	1.5	4.0	8.3	2.1
SI: *t*_*z*_ + 5 mm	3.8	6.2	1.6	4.2	8.1	2.3
SI: *t*_*z*_ + 10 mm	6.3	10.5	2.7	5.1	10.5	2.8

The initial rigid transformation aligns the chest wall of the corresponding prone-supine image pairs. For the landmark evaluation the reliable landmarks were used (c.f. rightmost three columns of Table 3)

## Conclusion

This paper presents for the first time a symmetric simulation based registration approach which accounts for large deformations present in prone-MRI-to-supine-CT breast image alignment. Our algorithm takes into account pre-loading of the breast geometry with gravity and calculates a virtually unloaded configuration. After an optimisation of soft tissues material parameters, the unloaded configuration is updated by accumulating image derived forces directly into the unloading simulation such that the unloaded configurations from prone and supine align. This results in a biomechanically constrained deformation. We enforce the motion on the chest wall to be parallel to the boundary between the breast and chest wall. Our novel unloading mechanism takes advantage of the duality between the forward simulation displacement description and the well established image resampling procedure which is inverse to the simulation.

To quantify the alignment accuracy, we performed measurements of the TRE in the central position by the means of manually picked landmarks. Although this is—due to the scale of the deformation at hand—an inherently difficult task, we measured a mean alignment error for all clinical cases of 5.6 mm.

Including prior knowledge of the gravity loading into the biomechanically based image registration was shown to be key to successful alignment. The pure biomechanical unloading step accounted for the biggest reduction of the overall TRE from 69.7 to 14.1 mm and the corresponding deformation recovery. The final corrections were image driven and smaller. They reduced the overall TRE from 14.1 to 5.6 mm.

Although the scheme proposed was implemented using the finite difference method, it can also be adapted to integrate the symmetric image derived forces into conventional finite element platforms.

The motion constraint presented here differs significantly from the frictionless sliding used for example by Han *et al.*^23^ and the fixed displacement constraint widely used elsewhere. Ultimately it allows control of sliding-like motion in a much more subtle way and could provide an experimental platform to investigate motion along the chest wall more precisely.

Having a supine target image is—especially in the context of image guided surgery—typically not routine clinical practice, and imposes a potential limitation on all intensity based registration methods for prone-to-supine breast image alignment. In the future we plan to explore methods which will work with more readily available target information, such as optical surface scans of the patient’s chest. This will open new applications in terms of image-to-physical registration.

The use of a mono-modal image force based on the sum of squared differences imposes a limitation on the framework which can be overcome by implementing image derived forces based on multi-modal similarity metrics such as normalised mutual information or other information theory based metrics. Possible implementations can be based on previous work, for instance that presented by Crum *et al.*^14^

## Figures and Tables

**Figure 12:**
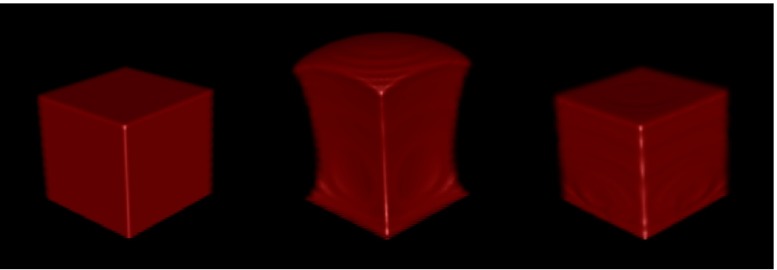
Loading and unloading experiment. The left box depicts the original unloaded configuration with dimensions $15 \times 15 \times 15{\text{mm}^{3}}$ which was exposed to gravity loading (central image). From this loaded configuration, the unloaded one was recovered, using our image based unloading mechanism.

## Appendix

### Unloading Evaluation

To test the newly developed unloading mechanism as presented in section “Gravity Unloading,” a simple mechanical loading-unloading experiment was conducted. A box geometry of size $15\times 15\times 15\,{\text{mm}^{3}}$, with a Young’s modulus of 500 Pa and a Poisson’s ratio of $\nu =0.45$ was applied with a body force and the material map resampled according to the forward simulation. Then, using the same material parameters and the warped geometry, the unloaded geometry was recovered using our proposed method. The results are shown in Fig. 12, where volume renderings of the material maps are presented. The left box depicts the original unloaded configuration, the central box the geometry after application of gravity, and the right box depicts the recovered unloaded configuration based on the central one. One can observe that the overall geometry was recovered well. Minor resampling artefacts can be observed. Since the unloading includes image processing steps, these artefacts can be attributed to the subsequent forward and backward resampling. Such artefacts however are not likely to be observable in the registration scheme, since repeated resampling is avoided in the symmetric design of the algorithm. Since the unloading is computed using a forward simulation only, the results can be compared directly. With a maximum displacement error of 0.6 mm and a 95-th percentile displacement error of 0.1 mm, the error is as expected of the order of the size of the image grid spacing, i.e., 0.5 mm.
